# An elevated likelihood of stroke, ischemic heart disease, or heart failure in individuals with gout: a longitudinal follow-up study utilizing the National Health Information database in Korea

**DOI:** 10.3389/fendo.2023.1195888

**Published:** 2023-08-23

**Authors:** Ho Suk Kang, Na-Eun Lee, Dae Myoung Yoo, Kyeong Min Han, Ji Yeon Hong, Hyo Geun Choi, Hyun Lim, Joo-Hee Kim, Ji Hee Kim, Seong-Jin Cho, Eun Sook Nam, Ha Young Park, Nan Young Kim, Sung Uk Baek, Joo Yeon Lee, Mi Jung Kwon

**Affiliations:** ^1^ Division of Gastroenterology, Department of Internal Medicine, Hallym University Sacred Heart Hospital, Hallym University College of Medicine, Anyang, Republic of Korea; ^2^ Hallym Data Science Laboratory, Hallym University College of Medicine, Anyang, Republic of Korea; ^3^ Division of Cardiology, Department of Internal Medicine, Hallym University Sacred Heart Hospital, Hallym University College of Medicine, Anyang, Republic of Korea; ^4^ Department of Otorhinolaryngology, Suseo Seoul E.N.T. Clinic, Seoul, Republic of Korea; ^5^ MD Analytics, Seoul, Republic of Korea; ^6^ Department of Medicine, Division of Pulmonary, Allergy, and Critical Care Medicine, Hallym University Sacred Heart Hospital, Hallym University College of Medicine, Anyang, Republic of Korea; ^7^ Department of Neurosurgery, Hallym University Sacred Heart Hospital, Hallym University College of Medicine, Anyang, Republic of Korea; ^8^ Department of Pathology, Kangdong Sacred Heart Hospital, Hallym University College of Medicine, Seoul, Republic of Korea; ^9^ Department of Pathology, Busan Paik Hospital, Inje University College of Medicine, Busan, Republic of Korea; ^10^ Hallym Institute of Translational Genomics and Bioinformatics, Hallym University Medical Center, Anyang, Republic of Korea; ^11^ Department of Ophthalmology, Hallym University Sacred Heart Hospital, Hallym University College of Medicine, Anyang, Republic of Korea; ^12^ Department of Pathology, Hallym University Sacred Heart Hospital, Hallym University College of Medicine, Anyang, Republic of Korea

**Keywords:** gout, stroke, ischemic heart disease, heart failure, cardiovascular diseases, longitudinal follow-up study, nationwide health insurance research database

## Abstract

**Objective:**

Accumulating evidence from other countries indicates potential associations between gout and cardiovascular diseases; however, the associations of gout with cardiovascular diseases, particularly stroke, ischemic heart disease, and heart failure, remain ambiguous in the Korean population. We hypothesized that individuals with gout are at a higher likelihood of stroke, ischemic heart disease, or heart failure. This study expands upon previous research by ensuring a comparable baseline between patient and control groups and analyzing 16 years of data derived from an extensive healthcare database.

**Methods:**

We selected 22,480 patients with gout and 22,480 control individuals from the Korean National Health Insurance Service-Health Screening Cohort database (2002–2019), and matched them at a 1:1 ratio according to sex, age, income, and residence. A Cox proportional hazard model with weighted overlap was employed to examine the relationship between gout and the risk of stroke, ischemic heart disease, or heart failure after adjustment for several covariates.

**Results:**

The incidences of stroke, ischemic heart disease, or heart failure in participants with gout were slightly higher than those in controls (stroke: 9.84 vs. 8.41 per 1000 person-years; ischemic heart disease: 9.77 vs. 7.15 per 1000 person-years; heart failure: 2.47 vs. 1.46 per 1000 person-years). After adjustment, the gout group had an 11% (95% confidence interval [CI] = 1.04–1.19), 28% (95% CI = 1.19–1.37), or 64% (95% CI = 1.41–1.91) higher likelihood of experiencing stroke, ischemic heart disease, or heart failure, respectively, than the control group.

**Conclusion:**

The present findings suggest that individuals with gout in the Korean population, particularly those aged ≥ 60 years, were more likely to have stroke, ischemic heart disease, or heart failure.

## Introduction

Gout, a serious systemic and metabolic disorder causing joint inflammation, has been demonstrated to be associated with high uric acid levels (1). The accumulation of monosodium urate crystals in the joints could contribute to the development of comorbidities, such as obesity, insulin resistance, hypertension, kidney problems, and hyperlipidemia (1, 2). Gout is more prevalent in males than in females; the overall prevalence is 2.9–4.5 and peaks in individuals aged ≥ 80 years (3, 4). In recent years, the prevalence of gout has increased worldwide owing to an aging population, obesity, and metabolic diseases (5, 6). The prevalence rate of gout increased 5.15-fold from 0.39% in 2002 to 2.01% in 2015 in Korea, which resulted in a yearly average rise of 10.8% in health insurance costs related to gout (7). This is a larger increase compared with that in other countries; the prevalence of gout in the UK, USA, and Taiwan increased 1.64-times between 1997 and 2012 (5), 1.4-fold between 1988–1994 and 2007–2008 (3), and 1.12-fold between 2005 and 2010, respectively (4, 8). Owing to the increasing elderly population and lifestyle changes that have occurred in recent years, gout and its related problems have emerged as a noteworthy health concern in Korea (7).

Cardiovascular diseases (CVDs) are the principal source of mortality globally, representing approximately one-third of all fatalities (9). Particularly, CVDs are the main cause of death in East Asian countries, such as Taiwan, Singapore, Japan, and Korea, behind cancer (9). In Korea, a majority (70%), a large proportion (41%), and nearly a fifth (19%) of the adults have at least one, two or more, and three or more risk factors for CVDs, respectively, such as hypertension, diabetes, hypercholesterolemia, obesity, and smoking (10). Findings from recent experimental and epidemiologic studies suggest an elevated risk of CVDs associated with gout (11–13), which has drawn particular attention. The breakdown of purines yields serum uric acid, resulting in an inflammatory state and heightened risk of CVDs (14–16). Inflammation caused by monosodium urate crystals could potentially activate inflammasome pathways, cause gout attacks in joints, and result in deposits of coronary plaques, thereby contributing to the excessive cardiovascular risk in gout (17, 18), similar to the mechanism of action of cholesterol crystals (19). In addition, research has identified a correlation between gout and an increased probability of myocardial infarction (20, 21), stroke (22, 23), and atrial fibrillation (24), all of which are part of the broader category of cardiac conditions (25, 26). A cohort study revealed that gout was associated with a 1.49-fold greater risk of overall CVDs (95% confidence interval [CI]=1.44–1.53), without considering potential confounding factors, such as lifestyle, body mass index (BMI), alcohol intake, and smoking (11). Moreover, a meta-analysis has demonstrated an association of uric acid-lowering agents with decreased risk of myocardial infarction (27). Since the elderly have more comorbidities, such as CVDs and metabolic diseases, the effects of these systemic disorders on CVDs are anticipated to become more prominent (1). Indeed, these CVDs caused by gout have emerged as a major public health concern, which needs to be urgently addressed to reach the health goal of disease prevention.

The incidence and mortality rates of stroke have decreased, while the mortality and hospitalization rates of heart failure and ischemic heart disease have increased in Korea (9). The trends may vary depending on the type of CVDs and gout (9). Nevertheless, few studies have investigated the potential relationship between gout and stroke, ischemic heart disease, or heart failure in the Korean population. Moreover, merely one study has explored uric acid-lowering agent-associated abnormalities in different CVDs, and the findings are conflicting (28). Examining the effect of gout on each CVD is essential since CVDs are a varied group of diseases with diverse causes but similar vascular risks (25). Therefore, additional studies considering the potential mutual confounding factors are necessary.

We hypothesized that individuals with gout might be more likely to have stroke, ischemic heart disease, and heart failure. This study builds on earlier work (28) by carefully balancing the baseline characteristics between the patient and control cohorts and examining 16 years of data compiled from a comprehensive healthcare database.

## Materials and methods

### Study population and participant selection

This study was approved by the ethics committee of Hallym University (2019-10-023), and the need for written informed consent was waived by the Institutional Review Board. All the analyses were performed in accordance with the rules and regulations of the ethics committee of Hallym University.

The Korean National Health Insurance Service-Health Screening Cohort (KNHIS-HSC) database provides Korean population-based and longitudinal information for research needs, which is selected in an arbitrary manner (29, 30). Since 1999, the KNHIS has been providing obligatory health insurance to nearly all Koreans. The KNHIS-HSC comprises anonymous and de-identified data and information. The diagnostic codes used in the KNHIS-HSC are based on the International Classification of Diseases, Tenth Revision, Clinical Modification (ICD-10-CM).

This longitudinal follow-up study retrospectively examined a gout group and a control group without a history of gout to determine the effect of gout on the probability of experiencing a stroke, ischemic heart disease, or heart failure. Participants with ICD-10 code M10 (gout) were identified from a total of 514,866 participants aged ≥ 40 years with 895,300,177 medical claim codes who had at least two clinic visits from 2002 to 2019 (n=27,313). To select participants who were diagnosed with gout for the first time, those who were diagnosed with osteoporosis in 2002 (1-year washout period, n=2470), did not have any documents of blood pressure level (n=1), or were diagnosed with stroke, ischemic heart disease, or heart failure before their gout diagnosis (n=2362) were all excluded.

Participants in the control group (n=487,553) did not match those in the gout group from 2002 to 2019, and 13,809 individuals with ICD-10 code M10 (gout) were excluded from the control group.

**Figure 1:**
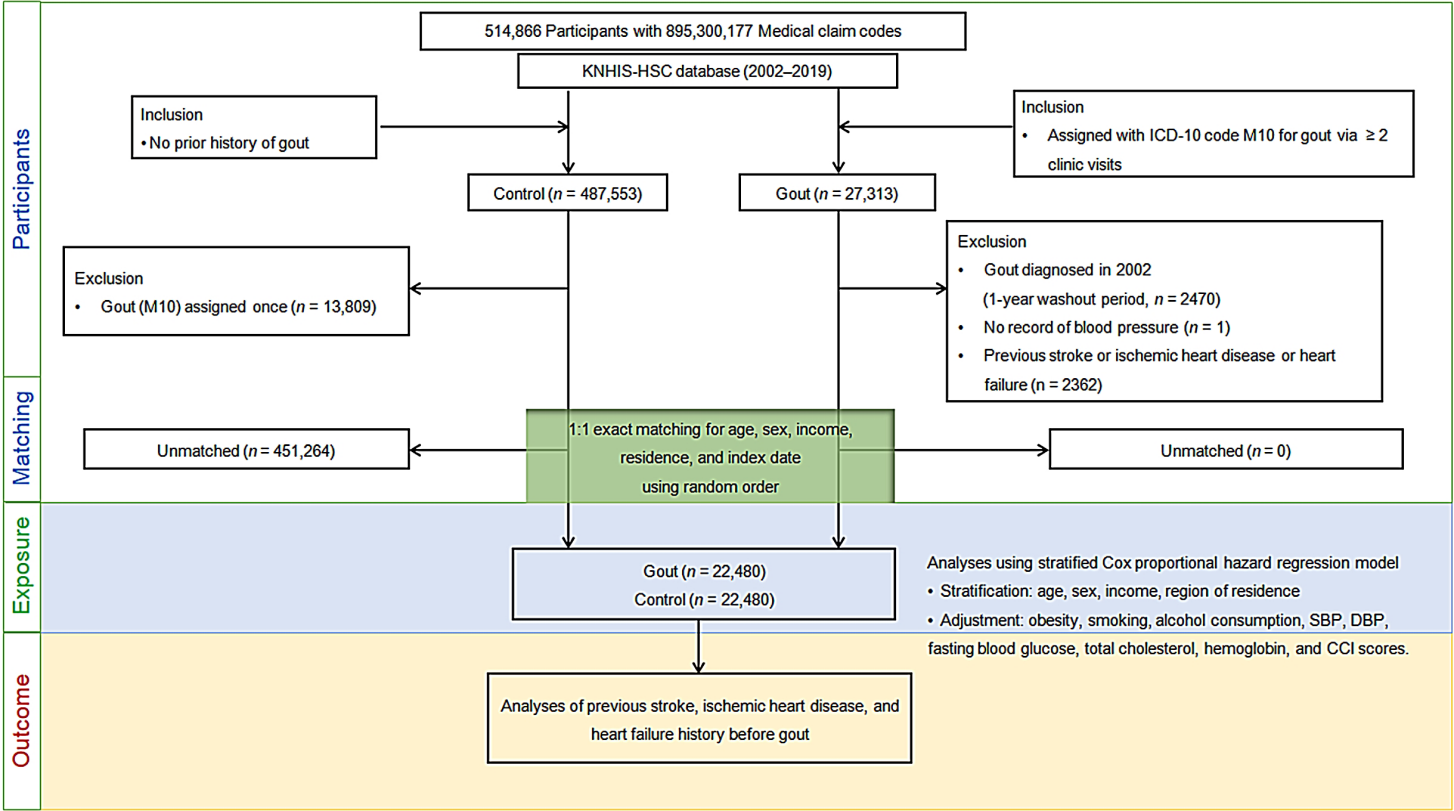
Flow of participant selection. Of the total 514,866 participants, 22,480 individuals with gout were paired with age, sex, financial status, and residential area-matched 22,480 controls. ICD-10, International classification of disease-10; CCI, Charlson comorbidity index; SBP, Systolic blood pressure; DBP, Diastolic blood pressure.

Matching was performed using a 1:1 ratio in terms of sex, age, economic status, and residence region to reduce variations in the basic demographic and medical characteristics between gout and comparison groups. To avoid selection bias, individuals without gout were randomly selected in numerical order. The index date for each participant with gout was the day when her/his gout diagnosis (M10) was initially logged in the medical insurance database. The index date for a control participant was the same as that for the matched participant with gout. All the participants in the comparison groups who died or had cardiovascular diseases before the index date were excluded. In total, 451,264 control participants were eliminated in the matching process. Finally, 22,480 participants with gout were paired with 22,480 control participants (**Figure 1**).

We examined the number of fresh diagnoses for stroke, ischemic heart disease, and heart failure based on ICD-10 labels in both gout and comparison groups from the index date to the end of 2019.

### Definition of gout

The study specified gout as a condition that had been either diagnosed or managed at least twice using ICD-10 codes (M10), as described previously (31, 32).

### Definition of cardiovascular disease

We included only the participants who had been hospitalized for at least two days or who had died owing to any of the following diseases: stroke (ICD-10 codes I60–I69), ischemic heart disease (I20–I25), or heart failure (I50). This selection criterion has been reported previously (33).

### Covariates

The 10 age groups ranged from 40–44 to ≥85 years in 5-year increments. Income classes were classified into 5 categories, with classes 1 and 5 having the lowest and highest salaries, respectively. The region of residence was classified as urban (Seoul, Busan, Daegu, Incheon, Gwangju, Daejeon, and Ulsan) and rural (Gangwon, Gyeongsangnam, Chungcheongbuk, Jeollabuk, Chungcheongnam, Jeollanam, Gyeongsangbuk, Gyeonggi, and Jeju) areas (34). In addition, participants were classified into current smokers, past smokers, or non-smokers based on smoking status. Alcohol consumption was divided into two categories based on consumption frequency (<1 time a week or ≥1 time a week). Obesity was classified into five categories as <18.5 (underweight), ≥ 18.5 to < 23 (normal), ≥ 23 to < 25 (overweight), ≥ 25 to < 30 (obese I), and ≥ 30 (obese II) using BMI (kg/m^2^) based on the Asia-Pacific criteria and the Western Pacific Regional Office 2000 (35).

Hemoglobin (g/dL), systolic blood pressure (SBP, mmHg), fasting blood glucose (mg/dL), diastolic blood pressure (DBP, mmHg), and total cholesterol (mg/dL) were also measured. The Charlson Comorbidity Index (CCI) was used to quantify the burden of 17 comorbidities (acute myocardial infarction, congestive heart failure, peripheral vascular disease, cerebral vascular accident, dementia, pulmonary disease, connective tissue disorder, peptic ulcer, liver disease, diabetes, diabetes complications, paraplegia, renal disease, cancer, metastatic cancer, severe liver disease, and HIV) (36, 37). The CCI calculated for these comorbidities was summed as the continuous variable (0 [no comorbidities] to 29 [multiple comorbidities]) (36, 37). Cerebrovascular disease, acute myocardial infarction, and congestive heart failure were excluded when calculating the CCI score (36, 37).

### Statistical analyses

Categorical data are expressed as percentages, and continuous data were summarized as means and standard deviations; the standardized difference was employed to compare the rate of general characteristics between the cohort sets. We employed propensity score overlap weighting to maintain the covariate balance and optimal sample size to diminish the probability of intergroup bias. We employed multivariable logistic regression to calculate the propensity score and subsequently used these scores to calculate the overlap weighting. The participants with gout were weighted using the probability of the propensity score, whereas the control participants were weighted with the probability of 1- propensity score, ranging from 0 to 1. The standardized differences before and after weighting were compared to examine the difference in general characteristics between gout and control groups. To assess the accuracy of matching, absolute standardized differences of the covariates before and after matching were compared, with ≤ 0.20 being considered an appropriate balance. The Cox proportional hazard model was used to adjust covariates with an absolute standardized difference of > 0.20 (38).

The Kaplan–Meier analysis and the log-rank test were employed to compare the collective risk of stroke, ischemic heart disease, and heart failure between gout and control groups. A Cox proportional hazard regression model with an overlap weight was used to determine the hazard ratios (HRs) and 95% CIs of gout in relation to stroke, ischemic heart disease, and heart failure. Both the crude (simple) and adjusted (for obesity, smoking, alcohol consumption, SBP, DBP, fasting blood glucose, total cholesterol, hemoglobin, and CCI scores) results were generated. The analyses were stratified based on factors, such as age, sex, income, and region of residence. Subgroup analyses were performed based on age (< 60 and ≥ 60 years) or sex (males and females). Statistical assessments were performed using two-tailed tests, and *p*-values of <0.05 were considered statistically significant. All the analyses were conducted using the SAS 9.4 software (SAS Institute Inc., Cary, NC, USA).

## Results

### Baseline characteristics

This study included 22,480 people diagnosed with gout between 2003 and 2019 and an equivalent number of age, sex, income, and region of residence-matched comparison participants. **Table 1** summarizes the baseline characteristics of both groups before and after an overlap-weighting adjusted PS matching procedure. The two groups were exactly alike in terms of demographic characteristics (standardized difference=0), except for the proportion of obese participants; the gout group had a higher percentage of obese participants than the control group (69.16% vs. 60.15%). However, after the overlap weighting adjustment process, the standardized differences of all the covariates were minimized, and the two groups were balanced (standardized difference ≤0.2).

**Table 1:** General characteristics of participants.

Characteristics	Before Overlap weighting adjustment			After Overlap weighting adjustment		
	Gout	Control	Standardized Difference	Gout	Control	Standardized Difference
**Age (years;mean, SD)**	61.0 (9.7)	61.0 (9.8)	0.00	61.0 (6.8)	61.0 (6.8)	0.00
**Age (years; n, %)**			0.00			0.00
**40–44**	590 (2.62)	590 (2.62)		286 (2.62)	286 (2.62)	
**45–49**	2058 (9.15)	2058 (9.15)		988 (9.06)	988 (9.06)	
**50–54**	3553 (15.81)	3553 (15.81)		1717 (15.74)	1717 (15.74)	
**55–59**	4571 (20.33)	4571 (20.33)		2219 (20.35)	2219 (20.35)	
**60–64**	3899 (17.34)	3899 (17.34)		1898 (17.41)	1898 (17.41)	
**65–69**	3144 (13.99)	3144 (13.99)		1531 (14.03)	1531 (14.03)	
**70–74**	2392 (10.64)	2392 (10.64)		1160 (10.64)	1160 (10.64)	
**75–79**	1436 (6.39)	1436 (6.39)		701 (6.43)	701 (6.43)	
**80–84**	648 (2.88)	648 (2.88)		315 (2.89)	315 (2.89)	
**85+**	189 (0.84)	189 (0.84)		90 (0.83)	90 (0.83)	
**Sex (n, %)**			0.00			0.00
**Male**	17,800 (79.18)	17,800 (79.18)		8,600 (78.85)	8,600 (78.85)	
**Female**	4680 (20.82)	4680 (20.82)		2,307 (21.15)	2,307 (21.15)	
**Income (n, %)**			0.00			0.00
**1 (lowest)**	3231 (14.37)	3231 (14.37)		1575 (14.44)	1575 (14.44)	
**2**	2747 (12.22)	2747 (12.22)		1334 (12.23)	1334 (12.23)	
**3**	3455 (15.37)	3455 (15.37)		1678 (15.39)	1678 (15.39)	
**4**	4750 (21.13)	4750 (21.13)		2295 (21.05)	2295 (21.05)	
**5 (highest)**	8297 (36.91)	8297 (36.91)		4024 (36.90)	4024 (36.90)	
**Region of residence (n, %)**			0.00			0.00
**Urban**	9563 (42.54)	9563 (42.54)		4643 (42.57)	4643 (42.57)	
**Rural**	12,917 (57.46)	12,917 (57.46)		6263 (57.43)	6263 (57.43)	
**BMI(kg/m^2^, mean, SD)**	24.8 (2.9)	23.9 (2.9)	0.29	24.4 (2.0)	24.3 (2.0)	0.06
**Obesity† (n, %)**			0.27			0.00
**Underweight**	302 (1.34)	567 (2.52)		194 (1.77)	194 (1.77)	
**Normal**	5663 (25.19)	7824 (34.80)		3246 (29.77)	3246 (29.77)	
**Overweight**	6221 (27.67)	6353 (28.26)		3106 (28.48)	3106 (28.48)	
**Obese I**	9328 (41.49)	7168 (31.89)		4006 (36.73)	4006 (36.73)	
**Obese II**						
**Smoking status (n, %)**			0.05			0.00
**Non-smoker or Past smoker**	17,390 (77.36)	17,862 (79.46)		8,559 (78.48)	8,559 (78.48)	
**Current smoker**	5090 (22.64)	4618 (20.54)		2,347 (21.52)	2,347 (21.52)	
**Alcohol consumption (n, %)**			0.10			0.00
**<1 time a week**	10,927 (48.61)	12,030 (53.51)		5571 (51.08)	5571 (51.08)	
**≥1 time a week**	11,553 (51.39)	10,450 (46.49)		5335 (48.92)	5335 (48.92)	
**SBP (mmHg; Mean, SD)**	129.42 (16.73)	126.94 (16.07)	0.15	128.15 (11.46)	128.15 (11.32)	0.00
**DBP (mmHg; Mean, SD)**	80.19 (10.97)	78.63 (10.41)	0.15	79.39 (7.50)	79.39 (7.33)	0.00
**Fasting blood glucose (mg/dL; Mean, SD)**	102.11 (27.52)	102.11 (29.65)	0.00	102.13 (19.97)	102.13 (19.93)	0.00
**Total cholesterol (mg/dL; Mean, SD)**	199.72 (39.83)	196.31 (37.48)	0.09	197.93 (27.24)	197.93 (26.45)	0.00
**CCI score (score; Mean, SD)**	0.84 (1.54)	0.72 (1.50)	0.07	0.78 (1.02)	0.78 (1.10)	0.00
**Hemoglobin (g/dL; Mean, SD)**	14.28 (1.51)	14.34 (1.43)	0.04	14.31 (1.05)	14.31 (1.01)	0.00
**Stroke (n, %)**	1666 (7.41)	1433 (6.37)	0.04	787 (7.22)	711 (6.52)	0.03
**Ischemic heart disease (n, %)**	1644 (7.31)	1219 (5.42)	0.08	773 (7.09)	608 (5.57)	0.06
**Heart failure (n, %)**	432 (1.92)	256 (1.14)	0.06	206 (1.89)	127 (1.17)	0.06

CCI, Charlson Comorbidity Index; SBP, Systolic blood pressure; DBP, Diastolic blood pressure; SD, standard deviation.

†Obesity was categorized as <18.5 (underweight), ≥ 18.5 to < 23 (normal), ≥ 23 to < 25 (overweight), ≥ 25 to < 30 (obese I), and ≥ 30 (obese II) based on body mass index (kg/m^2^).

### Incidence of stroke, ischemic heart disease, and heart failure in gout and control groups

In gout and control groups, stroke, ischemic heart disease, and heart failure occurred with incidence rates of 9.84 vs. 8.41, 9.77 vs. 7.15, and 2.47 vs. 1.46 per 1000 person-years, respectively; all the differences were statistically significant. The Cox proportional hazard analysis revealed that those with gout were 1.11, 1.28, and 1.64 times more likely to experience subsequent stroke, ischemic heart disease, and heart failure, respectively, than controls in the adjusted models (95% CI=1.04–1.19, p=0.003; 95% CI= 1.19–1.37, p<0.001; and 95% CI=1.41–1.91, p<0.001, respectively) over 16 years, after considering the demographic, lifestyle, and medical factors (**Table 2**).

**Table 2:** Crude and adjusted hazard ratios of gout for cardiovascular disease.

Dependent variable	IR per 1000 person-year		IRD per 1000 person-years (95% CI)	Hazard ratios for cancers (95% confidence interval)			
	Gout (n=3070)	Control (n=12,280)		Crude†	*p*-value	Adjusted†‡	*p*-value
**Stroke (n = 3099)**	9.84	8.41	1.43 (0.79–2.08)	1.17 (1.09–1.26)	<0.001*	1.11 (1.04–1.19)	0.003*
**Ischemic heart disease (n = 2863)**	9.77	7.15	2.62 (2.00–3.24)	1.36 (1.27–1.47)	<0.001*	1.28 (1.19–1.37)	<0.001*
**Heart failure (n = 688)**	2.47	1.46	1.01 (0.72–1.30)	1.69 (1.45–1.97)	<0.001*	1.64 (1.41–1.91)	<0.001*

IR, incidence rate; IRD, incidence rate difference; CI, confidence interval.

*Stratified Cox proportional hazard regression model, Significance at p < 0.05.

†Models were stratified by age, sex, income, and region of residence.

‡The Model was adjusted for obesity, smoking, alcohol consumption, systolic blood pressure, diastolic blood pressure, fasting blood glucose, total cholesterol, hemoglobin, and CCI scores.

**Figure 2:**
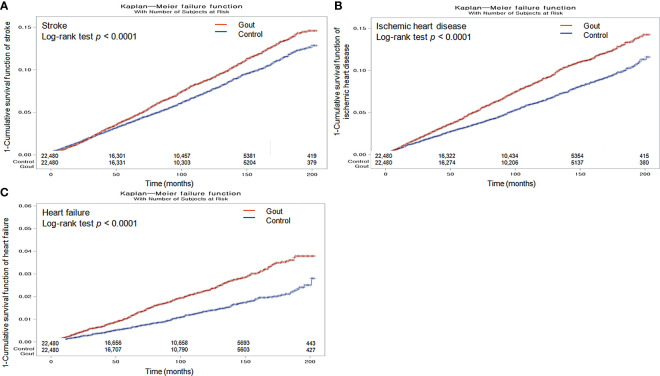
Kaplan-Meier probability of the incidence of stroke **(A)**, ischemic heart disease **(B)**, and heart failure **(C)** in gout and the control populations within 16 years of the index date.

The Kaplan–Meier analysis and the log-rank test indicated that individuals with gout had a greater likelihood of experiencing stroke, ischemic heart disease, and heart failure than those in the non-gout group (all p <0.0001; **Figures 2A–C**).

**Figure 3:**
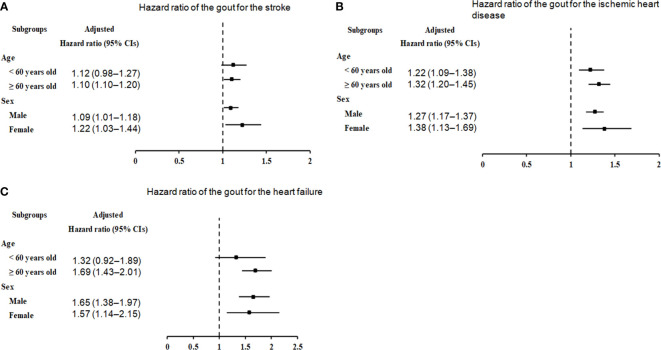
Forest plots for hazard ratios (95% confidence intervals [CI]) for the probability of stroke **(A)**, ischemic heart disease **(B)**, and heart failure **(C)** based on age and sex.

We further categorized the cohorts by sex and age to ascertain the potential relationship between gout and further incidence of stroke, ischemic heart disease, and heart failure. Stratification analyses showed that the threat of subsequent stroke was prominently augmented in participants with gout aged ≥ 60 years (HR=1.10, 95% CI=1.01–1.20, p=0.022), as well as in male (HR=1.09, 95% CI=1.01–1.18, p=0.036) and female (HR=1.22, 95% CI=1.03–1.44, p=0.018) patients (**Figure 3A**, **Supplementary Table 1**).

In the subgroup analyses by ischemic heart disease (**Figure 3B**, **Supplementary Table 2**), irrespective of age (< 60 years: HR=1.22, 95% CI=1.09–1.38, p<0.001; ≥ 60 years: HR=1.32, 95% CI=1.20–1.45, p<0.001) and sex (males: HR=1.27, 95% CI=1.17–1.37, p<0.001; males: HR=1.38, 95% CI=1.13–1.69, p=0.002), the correlation between gout and ischemic heart disease remained conspicuous.

In the subgroup analyses by heart failure (**Figure 3C**; **Supplementary Table 3**), the presence of gout was associated with an unremarkable but steady increase in the probability of heart failure in individuals aged ≥ 60 years (HR=1.69, 95% CI=1.43–2.01, p<0.001), as well as in men (HR=1.65, 95% CI=1.38–1.97, p<0.001) or women (HR=1.57, 95% CI=1.14–2.15, p=0.005).

## Discussion

Based on a large sample of Korean adults, this longitudinal study, considering the demographic data and pre-existing medical conditions, demonstrated that individuals with gout were slightly more likely to experience stroke, ischemic heart disease, or heart failure over the 16 years of observation. We identified a small but statistically-significant increased incidence of stroke, ischemic heart disease, and heart failure among individuals with gout compared with those without (stroke: 9.84 vs. 8.41 per 1000 person-years; ischemic heart disease: 9.77 vs. 7.15 per 1000 person-years; and heart failure: 2.47 vs. 1.46 per 1000 person-years). The weighted Cox proportional hazard analysis, adjusted for factors including age, sex, economic status, anemia, hypertension, hyperlipidemia, hyperglycemia, obesity, smoking, alcohol consumption, and comorbidities, also confirmed that individuals with gout were 11% (95% CI=1.04–1.19), 28% (95% CI=1.19–1.37), and 64% (95% CI=1.41–1.91) more susceptible to stroke, ischemic heart disease, and heart failure, respectively, than those without gout. This study with a long-term follow-up period provides further evidence that gout is an independent risk factor for CVDs, as demonstrated in a previous study (39). Thus, individuals with gout should be provided with extra information and training to ensure that they are aware of the potential risks of CVDs.

Our findings are consistent with those of a long-term survey that employed the National Health Insurance Research Database of Taiwan, which has a health insurance plan similar to that of Korea; 15,690 patients with gout and 246,210 controls were included in that survey, and the overall risk of heart diseases, including ischemic heart disease and heart failure, and the risk of stroke in the gout group were respectively 1.57 (95% CI=1.52–1.63) and 1.32 (95% CI=1.27–1.38) times higher than those in the control group (11). A study of 5713 Black and White men and women aged ≥ 65 years in the USA found a 1.97-fold greater risk (95% CI=1.22–3.19) of heart failure in the gout group than in the control group, but the risk of stroke was similar in both groups (odds ratio=0.83, 95% CI=0.48–1.43) (40). The discrepancies could be attributed to variations in the country settings, factors adjusted in the analysis, definitions of outcomes, underlying conditions, study sample, and populations involved in each study.

Stratification analyses by age and sex showed that men and women aged ≥ 60 years with gout were more likely to experience stroke, heart failure, and ischemic heart disease. However, the heightened risk of ischemic heart disease was evident regardless of age and sex; the finding indicates that gout might increase the risk of ischemic heart disease at an earlier age than that of stroke or heart failure, and patients with gout should be carefully monitored for ischemic heart disease. Moreover, this study demonstrated that males and females with gout had a similar risk of stroke (9% and 22% higher, respectively), ischemic heart disease (27% and 38% higher, respectively), or heart failure (65% and 57% higher, respectively) in the overall age group. Consistently, two Taiwanese studies also found that gout-related comorbidities were associated with an elevated risk of stroke in both sexes (22, 23). However, another Taiwanese study concluded that females had a significantly greater likelihood of overall CVDs than males, with an odds ratio of 2.06 (95% CI=1.94–2.20) for females versus an odds ratio of 1.26 (95% CI=1.21–1.31) for males (11).

Individuals with a tendency of increased alcohol intake, high BMI, elevated blood pressure, high total cholesterol level, proteinuria, and high uric acid levels in the Korean population are reportedly at a high risk of developing gout (1). The same risk factors shared between gout and CVDs might be a potential source of confusion. The pathophysiological associations of gout with CVDs are yet to be fully understood; however, genetic, lifestyle, and environmental factors, together with other unidentified elements, are presumably involved. Changes in the genes related to inflammasomes and their molecular associates could probably impact hyperuricemia and the risk of developing gout, as well as other conditions, such as metabolic syndrome and CVDs (41). Genome-wide association studies indicate that genetically predisposed elevated serum urate concentrations could potentially result in an elevated risk of gout, ischemic heart disease, or heart failure (26), with a notable percentage of cardiovascular risk being possibly derived from pleiotropic genes controlling xanthin oxidase activity and urate formation (26). Additionally, chronic inflammation and interleukin (IL)-1β pathways, as well as C-reactive protein, all of which are hallmarks of gout, may be associated with the pathogenesis of CVDs (42, 43). Upon leukocyte activation, endothelial dysfunction and other common pathogenic mechanisms, NLRP3 inflammasome activation, and IL-1β production are promoted in individuals with risk factors for CVDs (44–46). Moreover, research has corroborated that inflammation speeds up aging and therefore increases the chances of heart issues in gout, and telomere length, a risk factor for gout, is an autonomous element linked to the higher recurrence rate and CVD risk (12). Thus, upregulation of these inflammatory pathways in individuals with gout could, at least in part, explain the increased risk of CVDs.

The integrity of this study is primarily based on the comprehensive and nationwide population data that have been adapted to consider socioeconomic standing, potential lifestyle-related hazards, and associated health problems. Second, to limit selection bias and improve the precision of the study, two balanced groups (22,480 participants with gout and 22,480 participants without gout) were paired according to their likelihood of having gout, which could imitate randomized experiments. Despite the high prevalence of gout in men and the elderly, a balanced distribution of age and sex was possible by equally matching 22,480 individuals with gout against the same number of non-gout participants. The heterogeneity in terms of sex differences likely reflects distinctions in the original characteristics of the research groups (20). We found that both men and women with gout were more likely to suffer from stroke, ischemic heart disease, and heart failure. Third, the data gathered from all medical and clinic services in Korea enabled a compilation of complete medical histories throughout the study, thereby enhancing the generalizability and reliability of the research results. Additionally, this 16-year follow-up study is one of the most extensive longitudinal studies on the association between gout and CVDs.

Our findings should be interpreted with a few limitations in mind. First, because this study only included Korean nationals and relied on diagnosis codes, some confounding factors might not have been taken into consideration. Second, no data regarding the family history, personal genetics, or diets for gout or heart conditions were included in the KNHIS-HSC database. Creatinine, glomerular filtration rate, or urate lowering therapies or diuretics were not provided in this study (47, 48); thus, these data were not considered in this study.

In summary, our study demonstrated that individuals with gout in the Korean population, particularly those aged ≥ 60 years, were more likely to have stroke, ischemic heart disease, or heart failure. The findings suggest that individuals diagnosed with gout should receive additional information and training regarding the potential hazards of CVDs.

## Data availability statement

The original contributions presented in the study are included in the article/**Supplementary Material**. Further inquiries can be directed to the corresponding author.

## Author contributions

HK and N-EL: investigation, writing—original draft, and review & editing. MK: funding acquisition, writing—original draft, and review & editing. DY, KH, JH, HC, and HL: methodology. J-HK, JK, S-JC, and EN: formal analysis. HP: software. NK, SB, and JL: project administration. All authors have approved the final version of the manuscript and agree to be accountable for all aspects of the work. All persons designated as authors qualify for authorship, and all those who qualify for authorship are listed. All authors have read and agreed to the published version of the manuscript.
