# Supplementary material for: An elevated likelihood of stroke, ischemic heart disease, or heart failure in individuals with gout: a longitudinal follow-up study utilizing the National Health Information database in Korea
Source: Front Endocrinol (Lausanne). 2023 Aug 23;14:1195888. doi: 10.3389/fendo.2023.1195888 (PMC10482324; doi:10.3389/fendo.2023.1195888)
Supplement: Supplementary file 1 [file Table_1.docx]

Supplementary Material

An elevated likelihood of stroke, ischemic heart disease, or heart failure in individuals with gout: A longitudinal follow-up study utilizing the National Health Information Database in Korea

Ho Suk Kang ^1^†, Na-Eun Lee ^2^†, Dae Myoung Yoo ^2^, Kyeong Min Han ^2^, Ji Yeon Hong^3^, Hyo Geun Choi ^4^, Hyun Lim ^1^, Joo-Hee Kim ^5^, Ji Hee Kim ^6^, Seong-Jin Cho ^7^, Eun Sook Nam ^7^, Ha Young Park ^8^, Nan Young Kim ^9^, Sung Uk Baek^9^ , Joo Yeon Lee ^10^, and Mi Jung Kwon ^11,*^

† These authors contributed equally to this work and share first authorship.

*** Correspondence:** Mi Jung Kwon

Department of Pathology, Hallym University Sacred Heart Hospital, Hallym University College of Medicine, 22, Gwanpyeong-ro 170 beon-gil, Dongan-gu, Anyang-si, Gyeonggi-do 14068, Republic of Korea

(E-mail: [mulank@hanmail.net](mailto:mulank@hanmail.net) or mulank99@hallym.or.kr)

**S1 Table** Crude and adjusted hazard ratios of gout for stroke with subgroup according age and sex

|  | N of event /  N of total (%) | F/U duration  (PY) | IR per  1000 (PY) | IRD  (95% CI) | Hazard ratios | | | |
| --- | --- | --- | --- | --- | --- | --- | --- | --- |
|  |  |  |  |  | Crude | *p*-value | Adjusted† | *p*-value |
| **Age < 60 years old** | |  |  |  |  |  |  |  |
| Gout | 517 / 21,544 (2.4) | 100,613 | 5.14 | 0.94 (0.34–1.53) | 1.23 (1.08–1.39) | 0.002* | 1.12 (0.98–1.27) | 0.088 |
| Control | 426 / 21,544 (2.0) | 101,420 | 4.20 |  | 1 |  | 1 |  |
| **Age ≥ 60 years old** | |  |  |  |  |  |  |  |
| Gout | 1149 / 23,416 (4.9) | 68,655 | 16.74 | 2.14 (0.82–3.46) | 1.15 (1.05–1.25) | 0.001* | 1.10 (1.01–1.20) | 0.022* |
| Control | 1007 / 23,416 (4.3) | 69,003 | 14.59 |  | 1 |  | 1 |  |
| **Male** | |  |  |  |  |  |  |  |
| Gout | 1338 / 35,600 (3.8) | 135,962 | 9.84 | 1.23 (0.51–1.96) | 1.14 (1.06–1.24) | <0.001* | 1.09 (1.01–1.18) | 0.036* |
| Control | 1173 / 35,600 (3.3) | 136,287 | 8.61 |  | 1 |  | 1 |  |
| **Female** | |  |  |  |  |  |  |  |
| Gout | 328 / 9360 (3.5) | 33,306 | 9.85 | 2.23 (0.82–3.64) | 1.29 (1.10–1.52) | 0.002* | 1.22 (1.03–1.44) | 0.018* |
| Control | 260 / 9360 (2.8) | 34,136 | 7.62 |  | 1 |  | 1 |  |

 Abbreviation: IR, incidence rate; IRD, incidence rate difference; SBP, systolic blood pressure; DBP, diastolic blood pressure; PY, person-year;

* Stratified Cox proportional hazard regression model, Significance at *p*<0.05

† The model was adjusted for age, sex, income, region of residence, SBP, DBP, fasting blood glucose, total cholesterol, obesity, smoking, alcohol consumption, hemoglobin, and CCI scores.

**S2 Table** Crude and adjusted hazard ratios of gout for ischemic heart disease with subgroup according age and sex

|  | N of event /  N of total (%) | F/U duration  (PY) | IR per  1000 (PY) | IRD  (95% CI) | Hazard ratios | | | | | |
| --- | --- | --- | --- | --- | --- | --- | --- | --- | --- | --- |
|  |  |  |  |  | Crude | | | *p*-value | Adjusted† | *p*-value |
| **Age < 60 years old** | |  |  |  |  | | |  |  |  |
| Gout | 646 / 21,544 (3.0) | 99,496 | 6.49 | 1.60 (0.94–2.26) | | 1.33 (1.18–1.49) | | <0.001* | 1.22 (1.09–1.38) | <0.001* |
| Control | 493 / 21,544 (2.3) | 100,722 | 4.89 |  | | 1 | |  | 1 |  |
| **Age ≥ 60 years old** | |  |  |  | | |  |  |  |  |
| Gout | 998 / 23,416 (4.3) | 68,719 | 14.52 | 4.11 (2.93–5.28) | | | 1.39 (1.27–1.53) | <0.001* | 1.32 (1.2–1.45) | <0.001* |
| Control | 726 / 23,416 (3.1) | 69,688 | 10.42 |  | | | 1 |  | 1 |  |
| **Male** | |  |  |  | | |  |  |  |  |
| Gout | 1403 / 35,600 (3.9) | 134,726 | 10.41 | 2.69 (1.97–3.40) | | | 1.35 (1.24–1.46) | <0.001* | 1.27 (1.17–1.37) | <0.001* |
| Control | 1052 / 35,600 (3.0) | 136,147 | 7.73 |  | | | 1 |  | 1 |  |
| **Female** | |  |  |  | | |  |  |  |  |
| Gout | 241 / 9360 (2.6) | 33,489 | 7.20 | 2.32 (1.15–3.49) | | | 1.47 (1.21–1.79) | <0.001* | 1.38 (1.13–1.69) | 0.002* |
| Control | 167 / 9360 (1.8) | 34,263 | 4.87 |  | | | 1 |  | 1 |  |

 Abbreviation: IR, incidence rate; IRD, incidence rate difference; SBP, systolic blood pressure; DBP, diastolic blood pressure; PY, person-year;

* Stratified Cox proportional hazard regression model, Significance at *p*<0.05

† The model was adjusted for age, sex, income, region of residence, SBP, DBP, fasting blood glucose, total cholesterol, obesity, smoking, alcohol consumption, hemoglobin, and CCI scores.

**S3 Table** Crude and adjusted hazard ratios of gout for heart failure with subgroup according age and sex

|  | N of event /  N of total (%) | F/U duration  (PY) | IR per 1000 (PY) | IRD  (95% CI) | Hazard ratios | | | | | |
| --- | --- | --- | --- | --- | --- | --- | --- | --- | --- | --- |
|  |  |  |  |  | Crude | | | *p*-value | Adjusted† | *p*-value |
| **Age < 60 years old** | |  |  |  |  | | |  |  |  |
| Gout | 73 / 21,544 (0.3) | 102,810 | 0.71 | 0.23 (0.01–0.44) | | 1.47 (1.02–2.10) | | 0.037* | 1.32 (0.92–1.89) | 0.135 |
| Control | 50 / 21,544 (0.2) | 103,252 | 0.48 |  | | 1 | |  | 1 |  |
| **Age ≥ 60 years old** | |  |  |  | | |  |  |  |  |
| Gout | 359 / 23,416 (1.5) | 72,095 | 4.98 | 2.12 (1.48–2.77) | | | 1.75 (1.47–2.07) | <0.001* | 1.69 (1.43–2.01) | <0.001* |
| Control | 206 / 23,416 (0.9) | 72,098 | 2.86 |  | | | 1 |  | 1 |  |
| **Male** | |  |  |  | | |  |  |  |  |
| Gout | 326 / 35,600 (0.9) | 140,532 | 2.32 | 0.93 (0.61–1.25) | | | 1.67 (1.40–2.00) | <0.001* | 1.65 (1.38–1.97) | <0.001* |
| Control | 195 / 35,600 (0.5) | 140,426 | 1.39 |  | | | 1 |  | 1 |  |
| **Female** | |  |  |  | | |  |  |  |  |
| Gout | 106 / 9360 (1.1) | 34,373 | 3.08 | 1.34 (0.61–2.07) | | | 1.76 (1.29–2.42) | <0.001* | 1.57 (1.14–2.15) | 0.005* |
| Control | 61 / 9360 (0.7) | 34,924 | 1.75 |  | | | 1 |  | 1 |  |

 Abbreviation: IR, incidence rate; IRD, incidence rate difference; SBP, systolic blood pressure; DBP, diastolic blood pressure; PY, person-year;

* Stratified Cox proportional hazard regression model, Significance at *p*<0.05

† The model was adjusted for age, sex, income, region of residence, SBP, DBP, fasting blood glucose, total cholesterol, obesity, smoking, alcohol consumption, hemoglobin, and CCI scores.
